# A scoping review of post-earthquake healthcare for vulnerable groups of the 2023 Turkey-Syria earthquakes

**DOI:** 10.1186/s12889-024-18395-z

**Published:** 2024-04-02

**Authors:** Joseph Kimuli Balikuddembe, Jan D. Reinhardt, Ghanbari Vahid, Baofeng Di

**Affiliations:** 1 Institute for Disaster Management and Reconstruction, Sichuan University and Hong Kong Polytechnic University, Chengdu, Sichuan China; 2 Center on Disability Studies (CDS), University of Hawaii, Honolulu, Hawaii USA; 3 Swiss Paraplegic Research, Nottwi, Switzerland; 4 Department of Health Sciences and Medicine, University of Lucerne, Lucerne, Switzerland; 5 Rehabilitation Medicine Center, The first Affiliated Hospital of Nanjing Medical University, Nanjing, China; 6 Department of Nursing and Midwifery, Kermanshah University of Medical Sciences, Kermanshah, Iran; 7 Center for Archaeological Science, Sichuan University, Chengdu, Sichuan China

**Keywords:** Turkey*-*Syria earthquakes, Post-earthquake, Healthcare, Vulnerable groups, Scoping review

## Abstract

**Background:**

Identifying healthcare services and also strengthening the healthcare systems to effectively deliver them in the aftermath of large-scale disasters like the 2023 Turkey-Syria earthquakes, especially for vulnerable groups cannot be emphasized enough. This study aimed at identifying the interventions undertaken or proposed for addressing the health needs or challenges of vulnerable groups immediately after the occurrence of the 2023 Turkey-Syria earthquakes, as well as for prioritizing their healthcare service delivery in the post-Turkey-Syria earthquake.

**Methods:**

In this scoping review compiled with the five steps of the Arksey and O’Malley framework, five databases, including PubMed, Science Direct, Web of Science, OVID, and Google Scholar, were searched for studies published between March and April 2023 in line with the eligibility criteria. Interventions for enhancing post-earthquake healthcare services (PEHS) were grouped into seven (7) categories, adopted from previous guidelines and studies. Each one was assigned a default score of a value equal to one (1), which, in the end, was summed up.

**Results:**

Of the 115 total records initially screened, 29 articles were eligible for review. Different interventions they reported either undertaken or proposed to address the healthcare needs and challenges, especially faced by the most vulnerable groups in the aftermath of the Turkey*-*Syria earthquakes, were categorized into 7 PEHS. They were ranked with their scores as follows: humanitarian health relief (25); medical care (17); mental health and psychosocial support (10); health promotion, education, and awareness (9); disease surveillance and prevention (7); disability rehabilitation (7); and sexual and reproductive health (5).

**Conclusion:**

Since there are no proper guidelines or recommendations about the specific or most significant PEHS to prioritize for vulnerable groups after the occurrence of large-scale earthquakes, this scoping review provides some insights that can help inform healthcare service delivery and prioritization for vulnerable groups in the post-2023 Turkey-Syria earthquakes and other similar disasters.

## Introduction

The two earthquakes and their aftershocks struck southeastern Turkey and northern Syria on February 6th, 2023, at different time intervals and magnitudes of 7.8 and 7.6 on the Richter scale [1]. To date, they continue to arouse not only the bitter memories and tales of earlier and devastating earthquakes within Turkey and Syria and elsewhere, for example, the 1939 and Erzincan 2011 Van earthquakes in Turkey and the 1995 Gulf of Aqaba earthquake in Syria [2–4], but also left many survivors injured, disabled, and psychologically affected. They also tore apart families and communities, leaving hundreds of children orphaned or unable to be reunited with their parents and families [1, 4, 5]. By the end of April 2023, information from varying sources indicated that in both Turkey and Syria, the earthquakes had caused more than 56,000 deaths and 100,000 injuries and also partially damaged or completely razed over 230,000 buildings across the 11 provinces in Turkey, compared to 10,600 buildings in northwest Syria [1, 4]. This was unexceptional for various healthcare facilities like hospitals and health centers [4], yet their role was critically needed in not only saving the lives of people who were affected but also supporting a continuum of healthcare services during and in the post-2023 Turkey and Syria earthquakes. On top of that, the economic losses of the earthquakes were enormous. So far, the World Bank preliminary estimated the earthquake losses at US$34.2 billion and US$5.1 billion in Turkey and Syria, respectively, with nearly half of them attributed to direct damages to physical infrastructure [5].

Accordingly, the 2023 seismic events in Turkey and Syria (also known as the Kahramanmaras earthquakes) have been reported to be the deadliest earthquakes to hit the region in the past 20 years. They were, for instance, described as powerful as the 1939 Erzincan earthquake in Turkey, which caused over 32,000 and 100,000 deaths and injuries, respectively [4]. Ever since the twin earthquakes struck Turkey and Syria, the global community led by the United Nations (UN), through some of its specialized agencies like the World Health Organization (WHO) and the UN Office for the Coordination of Humanitarian Affairs (OCHA), in collaboration with the Turkish and Syrian governments and other non-governmental organizations (like SARD- Syrian Association for Relief and Development and Caritas International), has responded to the humanitarian plight of over 9 million people in either country [5, 6]. Notably, this was done by mobilizing monetary and non-monetary relief aid for addressing the urgent health and medical care needs for populations in the earthquake-affected areas in either country, for example, surgical care, maternal and pediatric care, emergency shelter, food and nutrition, proper water, sanitation, and hygiene (WASH), and disease prevention [4, 6].

Identifying different healthcare services and also strengthening the healthcare systems to effectively deliver them in the aftermath of large-scale disasters such as the 2023 Turkey-Syria earthquakes cannot be emphasized enough. This, however, ought to consider prioritizing the needs and services of vulnerable groups of the population, like children, women, elderly people, persons with disabilities (PwDs), persons with chronic diseases (PwCDs), refugees, or ethnic minorities. It is because they are most disproportionately affected and at high risk of witnessing diseases, injuries, premature deaths, and discrimination or abuse, as well as being disadvantaged and facing barriers to accessing essential healthcare services in the aftermath of disasters and emergency crises [7–9]. Arguably, this may be attributed to some conditions that are accentuated by their age, senility, disabling health conditions, gender, ethnicity, accommodation, education, geography, or political and socioeconomic-related hardships [8]. Thus, accurate data and information can play an instrumental role in informing decisions and choices for the timely allocation of limited or overstretched health resources, both for short- and long-term healthcare services. This is critically needed for settings like Turkey and Syria, which are already grappling with enormous political and socioeconomic challenges such as influxes refugees, population displacements, and fragile and overwhelmed healthcare systems, which were more likely to be worsened by the 2023 Turkey*-*Syria earthquakes. Therefore, the present scoping review aimed at identifying the interventions undertaken or proposed for addressing the health needs or challenges of different vulnerable groups immediately after the occurrence of the 2023 Turkey-Syria earthquakes, as well as for prioritizing their healthcare service delivery in the post-Turkey-Syria earthquake. The results herein are deemed timely and anticipated to help inform not only the seldom or limited research on subject matter but also policymaking on planning, prioritization, and allocation of resources for effective delivery of different healthcare services in the post-Turkey and Syria earthquakes and other similar and future large-scale disasters.

## Methods

### Study design

We anticipated the studies or literature about the recent 2023 Turkey*-*Syria earthquakes to be heterogeneous in nature and, on the other hand, limited or not yet comprehensively published. As such, the scoping review was considered to be an appropriate method for rapidly exploring the relevant interventions in the recently published studies that can enhance post-earthquake healthcare services (PEHS) undertaken or proposed in addressing the health needs and challenges for vulnerable groups following the 2023 Turkey*-*Syria earthquakes. This is because scoping reviews are less restrictive and are applicable for health research and studies, especially those for which the related or existing literature is initially uncertain [10, 11].

The current scoping review adopted the revised Arksey and O’Malley framework, which provides a foundation for scoping review methodology based on six (6) major steps [10]. The steps include: (a) defining the research question —that must be clearly defined to provide the roadmap for subsequent stages; (b) identifying relevant studies —involves identifying the relevant studies and developing a decision plan for where to search, terms to use, sources to be searched, time span, and language; (c) study selection —requires developing the inclusion and exclusion criteria based on the research question; (d) charting the data —involves developing a data-charting form for extracting data from sources; (e) collating, summarizing, and reporting results —helps to provide an overview of the breadth of the literature extracted but not a synthesis; and (f) consultation —which is optional and is used to involve stakeholder in suggesting additional references and providing insights beyond those in the literature. Two research questions informed this scoping review, as follows: (i) What interventions are reported in the recently published studies both undertaken and recommended to address the different healthcare needs of affected people immediately after the occurrence of the 2023 Turkey*-*Syria earthquakes? (ii) What interventions identified in recently published studies can be prioritized for healthcare service delivery, specifically targeting different vulnerable groups in the post-Turkey*-*Syria earthquake?

### Identification of interventions for post-earthquake healthcare services

Accordingly, there are no streamlined or context-specific recommendations for addressing PEHS for vulnerable groups. To this matter, to identify the PEHS for addressing their healthcare needs or challenges, we first reviewed the joint guidelines published by the WHO, World Bank, and European Union. The guidelines provide guidance to national and international stakeholders in the health sector about post-disaster needs assessments (PDNA) and recovery planning [12]. In addition, we also explored more information published about PEHS previously undertaken to assess the various needs of affected populations in the aftermath of disaster emergencies, for example, following the devastating Nepalese earthquakes in 2015 [13] and major earthquakes in China [14–16]. Moreover, two authors (JBK and RDJ) previously co-authored a study that explored the healthcare challenges after disasters in less developed countries and also recommended some measures for remedying them [17]. Considering those guidelines and studies, therefore, seven (7) healthcare interventions shown in Table 1 were identified as appropriate in responding to different health needs and challenges, specifically for vulnerable groups in the post-2023 Turkey*-*Syria earthquakes.

### Data sources

Between March and April 2023, we searched five electronic databases, namely PubMed, Science Direct, Web of Science, OVID, and Google Scholar, for relevant articles reporting different interventions both undertaken or proposed to address the health needs and challenges of people affected by the 2023 Turkey*-*Syria earthquakes. Based on the study objective and question, the two months were appropriate to explore and analyze the interventions, which were reported in emerging studies or literature to immediately be undertaken or recommended to address the healthcare needs of people, including different vulnerable groups affected by the 2023 Turkey*-*Syria earthquakes. The five databases were selected because they are comprehensive and freely searchable for academic literature, including those related to heterogeneous aspects of health.

### Search strategy

**Fig. 1:**
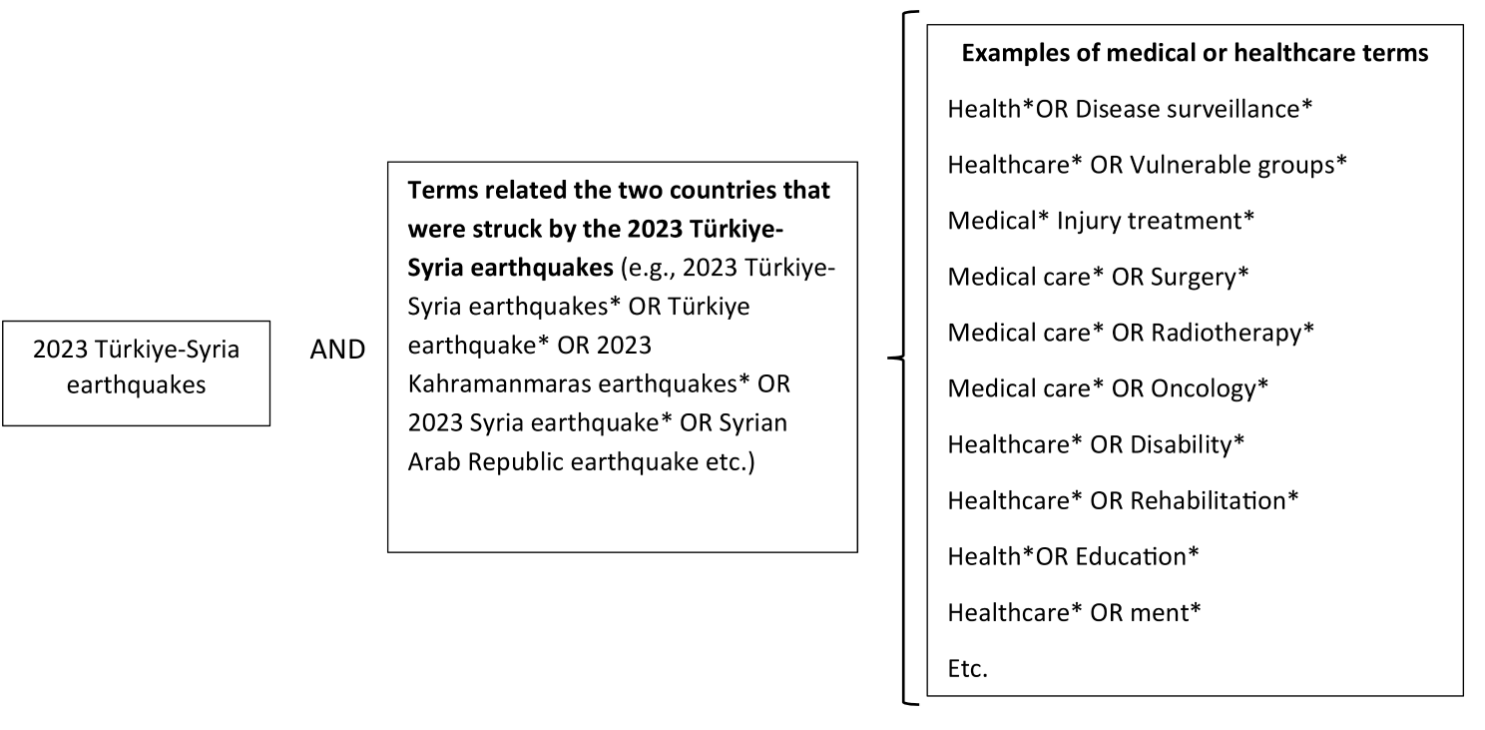
The search strategy that was followed in the scoping review

We developed the search strategy and search strings, drawing on our experience in conducting systematic reviews and meta-analyses, as can be attested to by some of our previously published studies, for instance, spanning patient prioritization and triage, injury, disability, and health-related rehabilitation [15, 18–21]. As indicated in Fig. 1, the search strategy was tailored to terms related or relevant to (1) the 2023 Turkey*-*Syria earthquakes; (2) two countries that were struck by the 2023 Turkey*-*Syria earthquakes; and (3) medical and healthcare services. Thus, different terms developed or identified were used by using the Boolean ‘OR’ and ‘AND’ operators, which we interchangeably combined during the search of five databases.

Initially, we had set out to search for only studies published within two months (7th February– 7th April, 2023) after the occurrence of the Turkey*-*Syria earthquakes. In the course of peer review, however, we deemed it necessary to expand the search to cover some more days or weeks up to the end of April (30 April, 2023). This intended to include the newly published studies as they slowly emerged. Moreover, reference lists of eligible studies were reviewed for additional studies to augment those identified in the initial search. In line with the search strategy, duplicates were removed from the updated searches against previous results to ensure records were accurate.

### Eligibility criteria and screening processes

We screened first the titles and abstracts to select studies that complied with the study aim and questions, followed by an evaluation of inclusion and exclusion criteria as summarized in Table 1. The first author (JBK) conducted the entire screening process independently to identify relevant studies, while the third and fourth authors (GV and BD) randomly selected more than half of the records and screened them independently. All four authors carefully read through the screened studies to identify and resolve any discrepancies through a detailed discussion and consensus. This also helped to avoid any screening bias that would arise from one reviewer. Afterwards, the two authors again cross-checked the titles and abstracts (if they were available) of the studies selected and agreed upon by the four authors to reach a final consensus on their eligibility. Full texts of eligible studies were later retrieved for data extraction. We anticipated that most of the evidence was likely to be from brief reports. As such, we intentionally adopted a more inclusive and less restrictive approach to include heterogeneous studies that are commonly excluded from the inclusion criteria of more rigid systematic review studies. In doing so, we intended to identify a wide range of interventions for delivering healthcare services in the context of earthquakes.

**Table 1:** Inclusion and exclusion criteria for screening of eligible studies

Inclusion criteria	
1	Studies in line with or focused on the subject matter
2	Studies in line with/related to the subject matter published only in English between 7th February and 30th April, 2023
3	Studies published as full-text articles
4	Studies that focused on different jurisdictions, both in Turkey and Syria, that were struck by the earthquakes
5	Peer-reviewed short studies involving reports, editorials, commentaries, perspectives, letters, opinions, news etc.
**Exclusion criteria**	
1	Studies that were out of context in line with the subject matter
2	Studies not published in English and also outside a period between 7th February and 30th April, 2023
3	Studies with their full-text were not accessible or available
4	Studies whose scope did not focus on the earthquake-affected areas both in Turkey and Syria
5	Reports of governments, UN agencies, non-governmental organizations and grey literature etc.

### Data extraction

The extraction of data from the studies was carried out by following a predesigned data extraction form generated in the Microsoft Excel Spreadsheet (version 16) a priori through discussion and consent among the authors. For quality appraisal, three authors reviewed the details that were extracted for the first 20% of studies. Lastly, the authors discussed the extracted results to ensure that they were consistent and in line with the aim and scope of the review. Thereafter, as the first author extracted details from the remaining studies, the other authors randomly validated their results to ensure that they were consistent. Quality assessment was not conducted since it is not a standard procedure for scoping reviews.

### Data analysis

Information of interest from retrieved studies was assembled for analysis using Microsoft Excel. This was followed by the critical and intensive line-by-line reading of the content of each study by at least two authors since the vast majority of studies were short papers. Analysis in this review was done, as aforementioned, based on the 4th and 5th (charting data, and collating, summarizing, and reporting results) of the Arksey and O’Malley framework [10]. However, this only focused on five steps, excluding the last one (consultation). At every step, discussions were virtually held among the authors to reach consensus on the information assembled in the Microsoft Excel form. The different interventions either undertaken or proposed to address various health needs or challenges aggravated by the 2023 Kahramanmaras earthquakes were aligned to their corresponding PEHS, whereby each one was assigned a default score of a value equal to one (1). In the end, the scores for each PEHs were summed up to determine their relevancy in supporting the delivery of healthcare services for different vulnerable groups in the post-2023 Turkey-Syria earthquakes. Information from retrieved studies was recorded and analyzed according to domains, including: (a) authorship, (b) date of publication, (c) country, (d) study type, (e) intervention(s) either undertaken or proposed, and (f) correspondence to PEHS for different vulnerable groups, as well as the score of each intervention for PEHS and study.

## Results

**Fig. 2:**
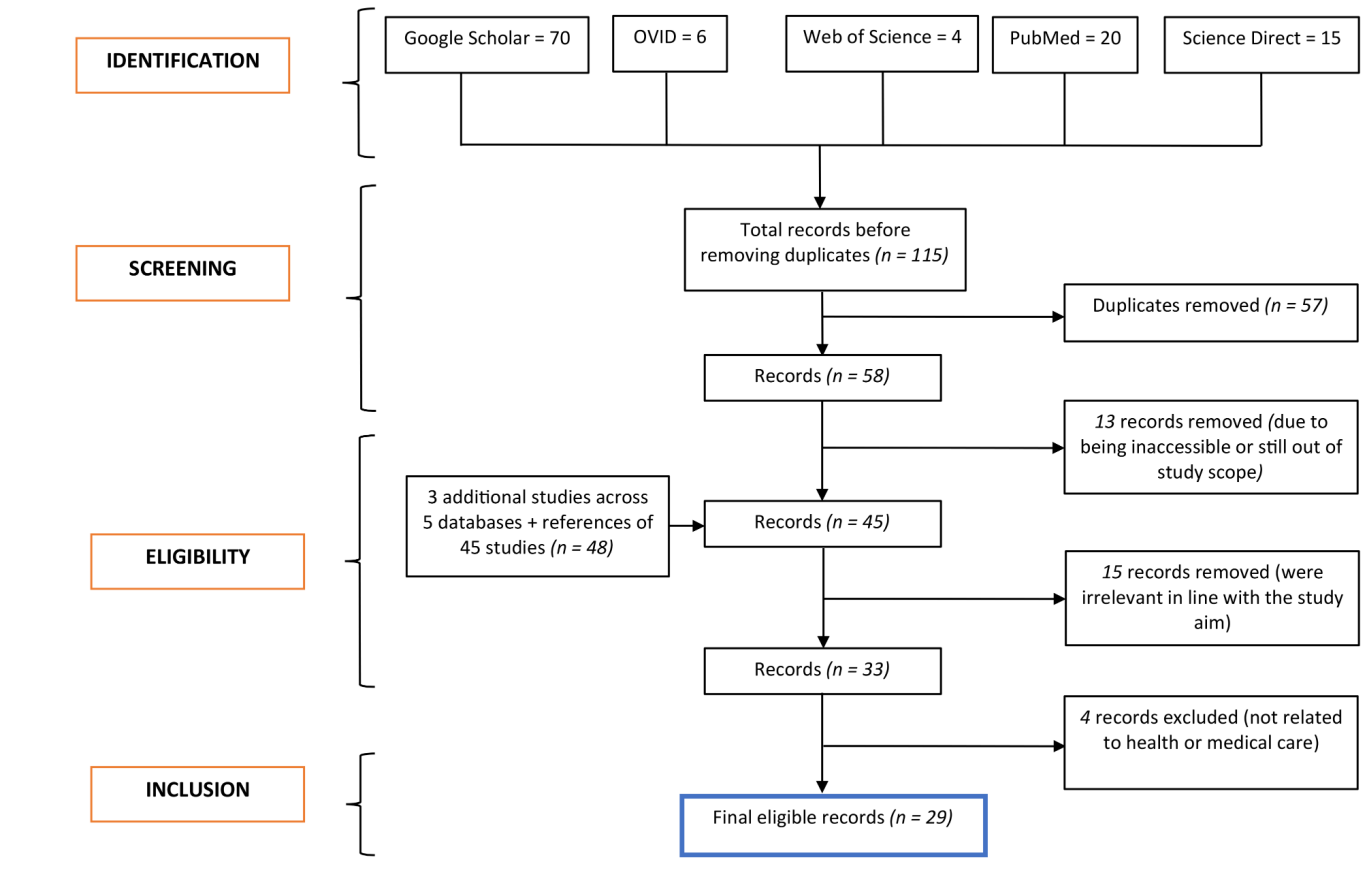
A flow chart illustrating the processes that were involved in this scoping review

Based on Fig. 2, the search yielded *115* studies or records, and *57* of them were removed after being assessed to be irrelevant to our scoping review or out of study scope. It should be noted that we only focused on screening the studies based on their titles since a large majority of them were simple or short studies without abstracts. As such, *58* records remained for screening against the eligibility criteria. Out of these, *13* studies were excluded mainly on the grounds of being unavailable, inaccessible, and again deemed out of study scope. Thereafter, we remained with *45* studies. In the hope of identifying more studies published after extending the study timeline from April 7th to April 30th, 2023, we still searched the five databases, as well as the references for 45 studies. This led us to only three additional records: 1 and 2 from references and Google Scholar, respectively, bringing the total to 48. We wish to note that, only 3 studies were added since most of them (which were still short or brief in nature) this time around focused on reporting the seismological characteristics of the earthquakes in either country. At this stage, we further screened them and found *15* duplicate studies that were also removed. The authors critically re-reviewed the remaining *33* records, and all agreed that four studies were irrelevant to both health and medical care and needed to be excluded. In the end, *29* published peer-reviewed records, which focused either on a single or two countries affected by the 2023 Turkey-Syria earthquakes and relevant to the study’s aim, were finally eligible for review. Figure 2 summarizes their searches, selection, and screening processes, whereas Table 2 presents some of their details as noted above.

### Study context and characteristics

In line with the review timeline, the retrieved studies were published as follows: February (*n =* 13), March (*n =* 14), and April (*n =* 2). Out of 29 records, a majority of them (*n =* 13) focused on Turkey [22–34], eleven (*n =* 11) on both Turkey and Syria [35–45], and six (*n =* 5) on Syria [46–50]. With regard to the publication type, most of the studies were published as news (*n =* 7) [24, 34, 35, 37, 38, 49, 50] followed by editorials (*n =* 6) [25, 28–31, 41], comments (*n* = 3) [33, 46, 47], correspondence (*n =* 3) [22, 32, 48], letters to the editor (*n =* 3) [27, 36, 44], and articles (*n =* 3) [39, 40, 45]. The rest of the studies were published under the categories of an image (*n =* 1) [23], opinion (*n =* 1) [42], perspective (*n =* 1) [43] and a brief world report (*n =* 1) [26]. Detailed designs were only specified in three articles, and they related to the use of an interactive tool to collect tweets from victims trapped in the rubble [39] and a synthetic aperture radar (SAR) to understand and evaluate the impact of the earthquake in southern and central Turkey, as well as northwestern Syria [40]. In the third article, post-earthquake surveys were conducted in the form of field visits to the most affected areas in southeastern Turkey and northern Syria [45]. Thus, it can be argued that within a period this scoping review was conducted, only three articles were retrieved with reported designs and also considered scientifically peer-reviewed.

### Vulnerable groups

The cross-cutting PEHS presented in the next sub-section were reported as both undertaken and proposed among the 29 studies to address the different healthcare needs and challenges faced by the most vulnerable and affected groups by the 2023 Kahramanmaras earthquakes. They specifically targeted the homeless people, children, women, girls, pregnant women, lactating mothers, older people, PwDs, PwCDs, Syrian refugees, and internally displaced persons (IDPs)–who mainly included the Kurdish minorities and people living or held in combatant zones of northwest Syria, as well as the affected rescue, medical, and healthcare workers [26–29, 34, 36, 37, 43, 47, 48]. These groups were targeted because, during and in the aftermath of large-scale disasters and emergencies like earthquakes, they are disproportionately affected, as well as overlooked in prehospital and hospital-based emergency preparedness and response plans [29].

## Post-earthquake healthcare services for vulnerable groups

The interventions we identified in the 29 studies relevant to addressing the healthcare needs or challenges of the above vulnerable groups in the post-Turkey-Syria earthquake are categorized into 7 PEHS. They are presented in Table 2 and later explained in subsequent sub-sections. The most significant interventions agitated for among the 29 studies, as far as the PEHS to address the healthcare needs and challenges for vulnerable groups or those with special needs who were at high risk or most affected by the 2023 Turkey-Syria earthquake are concerned, revolved around scaling-up their humanitarian health relief and medical care (*score = 25 and 17*, respectively). They were followed by those agitating for their mental health and psychosocial support (*n* = 10) and health promotion, education, and awareness (*score =* 9), as well as disease surveillance and prevention, and disability rehabilitation, which both registered the same score (*score =* 7). Sexual and reproductive health (SRH) registered the lowest score (*score =* 5).

**Table 2:** Selected details of the 29 final studies that were eligible for scoping review

					PEHS						
Authorship	Date	Country	Study type	Some intervention(s) undertaken or proposed	Medical care	Disease surveillance & prevention	Health promotion, education & awareness	SRH	MHPSS	Disability rehabilitation	Scaling-up humanitarian healthcare relief
Mahase E	Feb 7 2023	Turkey and Syria	News	Hospital evacuation, shipping blood stocks and donation, distribution of relief aids and medical supplies e.g., medicines, food, blankets etc.	1						1
Michael and Al-Jumaili	Feb 10 2023	Turkey and Syria	Letter to the Editor	Provision of medical supplies e.g., beta-blocker medications for hypertensive patients; health promotion and education related to medication adherence etc.	1		1				1
Onder et al.	Feb 15 2023	Turkey	Images in Medicine	Patient search, rescue and evacuation, radiological imaging, and accurate diagnosis and treatment etc.	1						
Sally H	Feb 15 2023	Turkey	News	Delivering food, water, health, nutrition, protection, shelter, and other lifesaving supplies to affected people; open the border into northwestern Syria for humanitarian aid to reach rebel-held areas; WHO distributed tons of medical and drug supplies; set up a mobile health clinic to provide specialized medical care and mental health etc.	1	1	1		1		1
Iacobucci G	Feb 15 2023	Turkey	News	Setting up polyclinics for outpatient treatment services focus on minor trauma, stabilisation, outpatient care, obstetrics and gynecology, pediatrics, and general practice; WHO launched a $43m (£36 m; €40 m) appeal to support the earthquake response etc.	1			1		1	1
Manjulika D	Feb 16 2023	Turkey and Syria	News	Provision of chemotherapy supplies and other medical facilities, as well as deployment of oncology teams etc.	1						1
Naddaf M	Feb 16 2023	Turkey and Syria	News	Retrofitting of building and enforcement of building standards	1						
Al-Droubi and Douglas	Feb 17 2023	Syria	News	Restoration of destroyed hospitals and health facilities; lifesaving surgeries and care of injured and those with disabilities; urgent response to containment of future disease outbreaks; provide WASH tools and services; international donation appeals, shipment of medicines and supplies etc.; health services for women and girls make up the majority of people taking refuge in shelters in north and northwest Syria; maternal health services and other programmes for women and girls have had to “scale up massively, etc.	1	1	1	1	1	1	1
The Lancet	Feb 18 2023	Turkey	Editorial	Shelter and psychological assistance; rebuild hospital and other collapsed infrastructure; orthopedic and brain surgeries, rehabilitation of affected medical staff (including National Medical Rescue Team members); intersectoral coordination i.e., excluding key stakeholders like military, doctors and engineers in crucial decision making etc.	1	1			1	1	1
Genc K	Feb 18 2023	Turkey	World Report	Collect demographic information from children and their families as soon as possible at the first point encountered for registration; address different issues affecting earthquake orphans, including organized sexual exploitation, etc.				1	1	1	1
Terzi and Dundar	Feb 21 2023	Turkey and Syria	Letter to the Editor	Development of an interactive tool that can provide situational awareness for missing and trapped people and disaster relief for rescue, treatment, and donation efforts, etc.	1						
Toraman et al.	Feb 26 2023	Tukey	Article	Provision of medical and surgical needs for children with severe trauma and/or crush syndrome; life-long physical rehabilitation for amputees; mental health care; and socioeconomic support, etc.	1				1	1	1
Düzova et al.	Feb 28 2023	Turkey	Editorial	A stepped-care approach to mental health service delivery during the recovery phase relying on callable mental interventions and services of both CMHWs with no formal mental health education and specialized mental health professionals; integrate psychology, psychiatry, and social work into the curriculum of mental health training and education, etc.			1		1		1
Kurt et al.	Mar 1 2023	Turkey	Correspondence	Consider specific surgical, medical, and rehabilitation needs and interventions for pediatric victims, who are often overlooked in prehospital and hospital-based emergency preparedness and response plans, etc.	1						1
Canpolat et al.	Mar 1 2023	Turkey	Editorial	Call for the support of nephrologists and their patients	1						1
Piccoli et al.	Mar 1 2023	Turkey	Editorial	Design, reinforcement, and construction of earthquake-resistant buildings in earthquake-affected areas, including hospitals and health facilities, etc.							1
Iftekhar A	Mar 2 2023	Turkey	Editorial	Funding, investments in infrastructure, equipment, and personnel, an adequate supply of medical supplies and equipment, improved communication, psychological support and interventions, etc.					1		1
Uwishema O	Mar 4 2023	Turkey and Syria	Correspondence	Continued search for missing people for treatment, restoration of collapsed buildings, etc.	1						
Gancheva and Dimova	Mar 4 2023	Turkey and Syria	Article	Psychological, social, and resilience support, and also teach victims to make themselves learn and practice protective measures, etc.			1		1		1
Najam and Sania	Mar 4 2023	Turkey and Syria	Editorial	Improve coordination and collaboration of relief efforts in resource mobilization between authorities and humanitarian organizations, provision of mental and psychosocial support, telepsychology interventions for injured and others affected, etc.						1	1
Cinar et al.	Mar 8 2023	Syria	Opinion	Scaling up humanitarian funding and ensuring resource availability for victims in northwest Syria; border opening; lifting and suspending some sanctions against Syria for 6 months, etc.							1
Alkhalil et al.	Mar 10 2023	Turkey	Comment	Extend the labyrinth corridor of the bunkers and remove the heavily shielded doors; modify the international recommendations and regulations in designing new bunkers in earthquake-prone regions; construct radiotherapy bunkers in separate buildings; establish a centralized online health database to protect patient files in case of massive destruction; promote a national and international collaboration network of radiation oncology centers and the radiotherapy workforce, etc.	1						1
Anacak et al.	Mar 10 2023	Syria	Comment	Increasing funding, providing more resources to local rescue and health responders; unimpeded cross-border access of government and rebel-controlled areas; a verifiable ceasefire; pushing stalled UN-brokered discussions on political settlement; launching an independent UN-mandated commission to investigate actions and inactions for earthquake response, etc.							1
Jabbour et al.	Mar 11 2023	Turkey and Syria	Comment	Humanitarian aid for affected populations, especially the most vulnerable populations such as children, pregnant women, people living with chronic health conditions or disabilities, older people, etc.							1
Villasana D	Mar 18 2023	Syria	Perspectives	Activation of the UN Central Emergency Response Fund and the UN Disaster Assessment Coordination Mechanism; earthquake aid transactions were fully exempt from sanctions for 180 days by the US Treasury, especially to help victims in rebel-held areas, etc.							1
Khaity et al.	Mar 18 2023	Turkey and Syria	Correspondence	Integrate MHPSS services into the primary healthcare system; establish mental health clinics; train mental health professionals; conduct community outreach programs; offer group sessions and creative therapies; provide self-care resources; develop a mental health hotline; promote education and awareness on mental health; train mental health professionals in trauma-related services; develop systems for monitoring the psychological health of survivors throughout the recovery process, etc.		1	1		1		1
Sirwan et al.	Mar 28 2023	Syria	Letter to the Editor	Search and rescue of mothers and babies; distribution of hygienic and dignity kits to women and families; supply medical equipment to two functioning hospitals; strengthen personnel and coordination on the ground, etc.		1	1	1			1
Duff E	April 1 2023	Turkey and Syria	News	Prioritize effective surveillance of emerging infections and potentially uncontrollable disease transmission at both local and regional levels, etc.	1	1	1				1
Mavrouli et al.	April 3 2023		Article	A frame of a multi-hazard approach to enhance effective surveillance of emerging infections and potentially uncontrollable disease transmission at both local and regional levels, etc.	1	1	1	1	1	1	1
**Total Score**					**17**	**7**	**9**	**5**	**10**	**7**	**25**

### Scaling-up humanitarian health relief

Several humanitarian measures divulged in the 29 studies argued for multisectoral coordination between political and humanitarian actors at the national and international levels to help people affected immediately and in the post-2023 Turkey-Syria earthquakes. They have advocated for the mobilization of funding and investment in rebuilding damaged hospitals and health facilities [26, 32, 50]. Also, are the provision, shipment, and donation of medical and drug supplies (e.g., blood stocks and donations, and beta-blocker medications for patients with cardiac and hypertensive complications); and the supply of medical equipment to functioning hospitals [35, 36, 49, 50]. Other humanitarian measures and calls aimed at mobilizing and strengthening the deployment of medical personnel and specialists on the ground (e.g., paediatricians, surgeons, cardiologists, oncologists, nephrologists, and radiotherapists) [28, 30, 33, 36, 37]. In particular, the WHO was reported to be instrumental in coordinating and reaching out to funders and stakeholders across the globe in appealing, mobilizing, and fundraising for donations to support different humanitarian programs [35, 48–50]. To this matter, a $43 million appeal was launched to support various humanitarian programs to respond to the medical and health needs of earthquake survivors [24]. Also reported were the humanitarian calls for the opening of unimpeded cross-border access in government and rebel-controlled areas, as well as the United States Treasury lifting and suspending some sanctions against Syria for at least six months to facilitate earthquake-related aid transactions [34, 50]. Ultimately, all these endeavors aimed at responding to the different needs of people affected by the 2023 Turkey-Syria earthquakes, including those for enhancing their health and wellbeing in the long-term.

### Medical care

Similar to some of the past and large-scale earthquakes like Bam 2003, Haiti 2010, Wenchuan 2008, Nepal 2015, and others [13, 15, 51, 52], the 2023 Turkey-Syria quakes have been reported to be associated with health effects ranging from disease outbreaks, injuries, trauma, and disability to mental and psychosocial complications [1, 4, 6]. As such, these conditions ought to be addressed by relying on inpatient and outpatient medical care for victims’ health and well-being, which can even last for days, months, or years. In this case, some of the notable interventions for inpatient and outpatient medical care identified in the 29 studies included patient evacuation to hospitals, radiological imaging, patient stabilization and treatment, lifesaving, orthopedic, and brain surgeries [23, 24, 26]. Others proposed were the dependency on specialized medical care and services that are rendered on both a short- and long-term basis and involved surgeries, obstetrics and gynecology, pediatrics, nephrology and dialysis, oncology, radiotherapy, and chemotherapy [24, 28, 30, 33, 36, 37]. As highlighted before, these services require competent medical specialists, who, among others, include pediatricians, cardiologists, oncologists, nephrologists, and radiotherapists. In the quest to adequately deliver those medical care services, special attention among the 29 studies was devoted to some special needs victims affected by the earthquakes, in particular pregnant women and lactating mothers, patients with cancer, hypertensive, cardiac, and kidney complications, and children with severe trauma and crush syndrome [26, 28–30, 33, 37, 43, 50].

### Mental health and psychosocial support

In this respect, mental health and psychosocial support (MHPSS) was revealed in the 29 studies for providing lifelong psychological, socioeconomic, and resilience support for victims of the 2023 Turkey-Syria earthquakes. MHPSS was proposed to target the affected victims with post-mental health and psychosocial symptoms and conditions such as post-traumatic stress disorders, stigma, anxiety, and depression [22, 28, 32, 41, 44]. Among the MHPSS services proposed included telepsychology [42]; training community mental healthcare workers (CMHWs) and other professionals; and also providing self-care resources, creative and group-based mental therapeutic sessions, and community mental outreach programs [44]. These MHPSS services were argued to be integrated into the primary healthcare systems of Turkey and Syria and also be augmented by setting up mobile health clinics to help in providing specialized mental healthcare and psychological assistance [22, 44], especially to the most vulnerable groups such as the injured and disabled, homeless, and unaccompanied children, as well as HCW and medical rescue personnel who were physically and emotionally overwhelmed and at high risk of anxiety [26, 27]. MHPSS services were proposed to be delivered by different professionals, such as psychologists, psychiatrists, and social workers [41, 44].

### Disease surveillance and prevention

Some measures for averting the potential risk of communicable and non-communicable diseases, which are inevitable in the aftermath of devastating disasters like the 2023 Turkey-Syria earthquakes, were disclosed in the 29 studies. The measures involved the distribution of WASH (water, sanitation, and hygiene) kits to women and girls, who comprised the majority of Kurdish minority refugees in the camps in northwest Syria, to support their hygienic dignity [26, 50]. Also reported were the provision of ready-to-eat hot food, as well as sheltering tents, heating, and thermal blankets for homeless people and those sleeping in streets, malls, schools, mosques, or open spaces that exposed them to freezing temperatures and cold weather, which are risk factors for respiratory infections and hypothermia [26, 34, 35, 49, 50]. On the other hand, one of the interventions called for the populations in earthquake-affected areas in southeastern Turkey to avoid uncontrolled waste disposal sites and stagnant water. This is because it could expose them to waterborne infections such as cholera and diarrhea, which commonly emanate from the consumption of unclean and contaminated surface water, soil, and animal urine [50]. Above all, the importance of prioritizing and developing effective surveillance systems for monitoring diseases and the psychological health of survivors throughout the recovery process was also underlined [44]. This was intended to support the early detection of disease symptoms so that appropriate mitigation, diagnosis, and treatment interventions to prevent their morbidity and mortality could be undertaken.

### Health promotion, education, and awareness

Across the 29 studies, the programs for promoting education and awareness on various aspects of health among the survivors of the 2023 Turkey-Syria earthquakes were disclosed. Some of them revolved around sensitizing populations on how to contain future disease outbreaks and transmissions [50]; teaching the victims some of the protective and resilience practices during and in the post-earthquakes [41]; training HCWs on how to encourage their patients, especially PwCDs, to adhere to medication [36]; and community outreaches to raise awareness on ill mental health and its associated impacts [44]. Further programs advocated for the integration of psychology, psychiatry, and social-related works into the curriculum of mental health training and education, as well as the training of mental health professionals in trauma-related services. The training was encouraged to be offered prior to deploying the trainees in earthquake-affected areas to offer necessary support to earthquake victims exhibiting different mental health symptoms or effects. Also, some programs advocated for organizing sessions for mental health therapies to allow individuals with mental problems to share and discuss their life experiences, connect with their peers also affected by the earthquakes, and seek treatment to overcome their mental health effects [22, 44]. Ideally, all these health education and awareness interventions were recommended to be rendered through public events, social media campaigns, or educational materials. Their delivery, moreover, was recommended to involve stakeholders both with and without formal health professional training and education [22, 41], for instance, community leaders, CHCWs, psychologists, psychiatrists, and social workers.

### Disability rehabilitation

A few interventions were still identified among the 29 studies to target minimizing of loss of physical functioning, restoring functional ability, and increasing or maintaining the current functioning of the victims who were injured, traumatized, and disabled as a result of the 2023 Turkey-Syria earthquake. In regard to this, outpatient rehabilitation care and treatment, as well as life-long physical rehabilitation, were proposed and offered by some service providers, like orthopedic and brain surgeons [23, 24, 26]. They were largely proposed to focus on the injured, wounded, and amputated victims who sustained minor and severe injuries, trauma, crush syndrome, and rhabdomyolysis [23, 24, 28, 43]. Rehabilitation was also recommended for persons with existing disabilities or injured adults, unaccompanied children who were separated from their families, and also for medical and rescue workers who were injured in the course of engaging in rescuing the victims trapped in collapsed building rubble [23, 24, 26–29, 42, 50].

### Sexual and reproductive health

Similar to disability rehabilitation, fewer of the 29 studies divulged on programs for supporting SRH among victims of the 2023 Turkey-Syria earthquake, especially for women and girls, who make up the majority of the Kurdish ethnic minorities displaced and refugees in refugee camps in northwest Syria. The programs aimed at rendering outpatient obstetric and gynecological care, scaling up maternal health services for girls, pregnant women, and lactating mothers, and offering basic hygiene products and care kits to women and girls to enhance their hygienic dignity [24, 26, 49, 50]. Another SRH program, on the other hand, was intended to mitigate organized sexual exploitation that was likely to be faced by the “earthquake orphans” [27].

## Discussions

Delivering appropriate healthcare services to the most vulnerable groups of the population in the immediate and aftermath of large-scale disasters may be challenging. One of the 29 studies attributed it to the fact that, oftentimes, some vulnerable groups are overlooked in the planning of prehospital and hospital-based emergency preparedness and responses [29]. Moreso, the two authors (JBK and RDJ) in their study about healthcare challenges after disasters in lesser developed countries decried the limited consideration and priority in existing disaster legislation and response plans given to the special needs of vulnerable populations [17]. As such, delivery of different healthcare services, especially to the most vulnerable groups of people affected by mega earthquakes now and in the future of similar magnitude of the 2023 Turkey*-*Syria quakes, will likely be complicated by a lack of proper guidelines or recommendations about the specific or most significant healthcare services to prioritize, in particular, when the demand for available resources is overwhelming.

As aforementioned, the 2023 Turkey*-*Syria earthquakes led to devastating impacts, such as mortalities and injuries, population displacements, and massive damages to buildings [1, 5, 42, 53]. While some of these impacts have continued to manifest, others are highly anticipated to be witnessed in the coming months or in the near future [42, 53]. Without adequate plans for responding to them, any emerging or future impacts associated with the 2023 Kahramanmaras earthquakes will impose grave effects on the most vulnerable groups of the population. This is because, they are not only disproportionately affected by disasters and emergency crises, but are also at higher risk of witnessing their health effects [7–9, 17]. It is, thus, not surprising that the call for scaling up humanitarian healthcare relief for the affected people of the Turkey-Syria earthquakes dominated the other interventions in a majority of 29 studies. Indeed the call was timely, if we can contemplate what would be the short- and long-term plight of vulnerable groups like cancer patients, children, pregnant and breastfeeding women, Syrian refugees, Kurdish IDPs, and ethnic minorities following the occurrence of the Turkey*-*Syria earthquakes in February 2023 [26–29, 34, 36, 37, 43, 47, 48].

On top of that, vulnerable groups face a higher risk of experiencing the diseases, injuries, ill mental conditions, and, above all, premature deaths they aggravate during and in the aftermath of disasters or emergency crises [8, 9, 17]. Whenever disasters occur, they disrupt the functioning of healthcare facilities and halt the accessibility and delivery of critical healthcare services that people with special health needs rely on. This has not been unexceptional with the recent 2023 Turkey*-*Syria earthquakes, in regards to PwCDs and others experiencing various disabling health conditions. Notably, the earthquakes disrupted the chemotherapy, radiotherapy, brachytherapy, and renal dialysis services —which are heavily relied on by cancer and kidney patients for their treatment both in Turkey and Syria [30, 33, 37]. Also, the quakes were reported to have likely impeded cardiac, hypertensive, and diabetic patients from accessing essential medicines and supplies, which could end up reducing their adherence to medications [25, 36].

Additionally, some of the HCWs and first responders who were critical in administering care to PwCDs and other patients, as well as at the frontlines of rescuing and saving lives, were also not spared from the wrath of the Turkey*-*Syria earthquakes. Some were either rendered homeless, injured, killed, or even lost their property and loved ones [22, 33, 35, 37]. In this case, having safe and resilient healthcare systems in place that are always ready to deliver quality healthcare services across the continuum and guarantee the safety of patients and HCWs during and after mass-causality earthquakes and other disasters is critically important. Therefore, the healthcare systems both in Turkey and Syria ought to be strengthened so that they are better prepared to effectively respond to future crises, maintain core functions when they are affected by crises like the 2023 devastating earthquakes, and, above all, be re-organized based on lessons learned during the crises [54].

Like some of the past devastating earthquakes (e.g., Bam 2003, Haiti 2010, Wenchuan 2008, Nepal 2015, and others [13, 15, 51, 52]), there’s high anticipation that a large proportion of victims who were severely injured, displaced, lost their property and loved ones, and separated from their families or related social networks due to the 2023 Turkey*-*Syria earthquakes will present ill mental health conditions [55]. Already, some of them, like post-traumatic stress disorders, stigma, anxiety, and depression, were reported to be exhibited among HCWs and first responders, and a vast majority of Syrian refugees and IDPs, currently estimated to be over 15 million and for over a decade, have witnessed dire humanitarian challenges as a result of the protracted Syrian conflicts [22, 44]. As such, the MHPSS compounded with disability rehabilitation, as revealed across the 29 studies can be utilized as some of the most effective interventions for restoring optimal health, physical functioning, autonomy, and activity of daily living. This is not only needed for the Syrian refugees and IDPs but also for HWCs, PwDs, and PwCDs, who are no doubt at great risk of suffering from mental illness and psychiatric-related disorders induced by the impacts of the 2023 Turkey*-*Syria earthquakes. Moreover, MHPSS could be embraced to promote not only their mental health and psychosocial wellbeing but also their short- and long-term rehabilitation and recovery, as well as enhancing their subsistence livelihood and inclusion in community support networks [18, 56–59].

However, MHPSS interventions along with disability rehabilitation will not alone be enough to enhance the health improvement and wellbeing of the victims of the 2023 Turkey*-*Syria earthquakes. Instead, they ought to be implemented hand in hand with SRH and educational programs aimed at sensitizing and raising awareness among the populations at high risk of disease outbreaks and also acquainting them with knowledge and skills about the necessary actions for reducing and preventing them from spreading in the aftermath of earthquakes [60]. This deserves to pay special consideration, especially for the most vulnerable groups, in particular women and children, who oftentimes are the first victims to witness or experience disasters and their impacts [61]. Fortunately, some measures for promoting both SRH and disease control, like the avoidance of uncontrolled waste disposal and stagnant water, as well as the distribution of WASH kits and shelter items, were disclosed across the 29 records [26, 50]. Others advocated for developing and strengthening the surveillance systems for monitoring diseases and the psychological health of survivors throughout the recovery process of the 2023 Turkey and Syria earthquakes [45]. No doubt, these measures can play a critical role in mitigating water- and air-borne diseases like respiratory infections, hypothermia, cholera, and diarrhea [26, 28], which commonly prevail among displaced and homeless people, refugees, IDPs, children, women, and girls in earthquakes and other large-scale disasters.

## Study limitations and conclusion

The results and discussions presented herein provide insights that help inform the planning for delivery of healthcare services, especially for the most vulnerable groups of the populations in the post-Turkey and Syria earthquakes. However, the findings of this review should be cautiously interpreted, especially in informing policymaking since, apart from 3 studies, the rest were not scientific in terms of having ad hoc research objectives, methods, appropriate sample sizes, and study settings. Another noteworthy concern is that our scoping review may have missed out on other eligible studies based on its criteria of considering only studies published in English or having some of their content translated in English. Also, the reports and other documents published by various stakeholders were not considered in this review. Yet, even if they have not been formally evaluated or published, they would still have provided additional information for informing PEHs about addressing the different healthcare needs and challenges of vulnerable groups and general populations affected by the 2023 Turkey*-*Syria earthquakes. These limitations notwithstanding, it is vital to note the time and circumstances in which the 29 studies were published. Instead, they are commendable for providing a snapshot of the interventions that were promptly undertaken or proposed to address the healthcare needs of populations affected by the 2023 Turkey*-*Syria earthquakes. Nonetheless, the 29 studies laid a foundation for future and comprehensive research on cross-cutting PEHS for vulnerable groups in the post-Turkey*-*Syria earthquakes and other similar earthquakes, and our scoping review found to revolve mainly on humanitarian health relief and medical care among the 7 interventions.

## Data Availability

The datasets used and/or analyzed during this study is available from the corresponding author on reasonable request.
